# Disseminated gonococcal infection during two decades in the university hospital, Thailand

**DOI:** 10.2478/abm-2024-0018

**Published:** 2024-06-28

**Authors:** Kanphai Wongjarit, Sittichai Ukritchon

**Affiliations:** Department of Microbiology, Faculty of Medicine, Chulalongkorn University, Bangkok 10330, Thailand; Division of Rheumatology, Department of Medicine, Faculty of Medicine, Chulalongkorn University, Bangkok 10330, Thailand

**Keywords:** antimicrobial susceptibility, clinical features, complications, disseminated gonococcal infection, gonococcal arthritis, *Neisseria gonorrhoeae*

## Abstract

**Background:**

Disseminated gonococcal infection (DGI) caused by *Neisseria gonorrhoeae* commonly presents with the classic triad of polyarthritis, tenosynovitis, and dermatitis. There is no clinical and microbiological data of DGI in Thailand.

**Objective:**

To study the clinical features, outcomes of treatments, and antimicrobial susceptibility data of DGI patients.

**Methods:**

All medical records of DGI patients at King Chulalongkorn Memorial Hospital (KCMH) from January 2002 through September 2019 were reviewed and analyzed. The patients were defined as definite DGI (the clinical features and the evidence of gonococcal infection) and probable DGI (clinical features with response to treatment with third-generation cephalosporins and with no evidence of gonococcal infection).

**Results:**

There were 41 patients (27 definite and 14 probable DGI), with a male-to-female ratio of 1:1.4 and median age of 30 years. The middle-age and elderly group accounted for 20% of the patients. The clinical features were fever (90.27%), arthritis (92.7%), tenosynovitis (63.4%), and genitourinary symptoms (29.3%). The most common pattern of joint involvement was oligoarthritis (52.6%). The majority of the patients had good clinical outcomes, while complications occurred in 4.8% of the patients including osteomyelitis and pyomyositis. All 19 antimicrobial-susceptibility results were susceptible to ceftriaxone.

**Conclusions:**

During the past 2 decades in KCMH, the age of the DGI patients tends to be older, and there is no gender difference as in the historical studies. The clinical features are still similar to the previous studies. The majority of the patients had good clinical outcomes. There is no case of ceftriaxone-resistant *N. gonorrhoeae*.

Gonorrhea is a common sexually transmitted infection (STI), caused by *Neisseria gonorrhoeae*, that typically affects the mucosal surfaces [1]. In some circumstances, the pathogen can invade into the bloodstream and spread to the other organs causing disseminated gonococcal infection (DGI) with the classic triad of arthralgia or arthritis, tenosynovitis, and dermatitis [2], [3], [4]. Arthralgia or arthritis is a clinical spectrum of DGI, which accounts for >85% of the cases [5], [6], [7], [8]. Previous studies in King Chulalongkorn Memorial Hospital (KCMH) reported that gonococcal arthritis was responsible for 8.1% and 1.9% of patients with bacterial arthritis during 1976–1985 and 2006–2010, respectively [9, 10]. To date, no previous publication has described the clinical features of Thai patients with DGI.

According to the annual epidemiological surveillance report of the Thailand Department of Disease Control, the gonorrhea incidence rate increased from 8,544 cases in 2015 to 9,243 cases in 2016, which raised awareness of the reemergence of the disease [11]. However, there was no report on the incidence rate of DGI and gonococcal arthritis, which remains to be elucidated. Ceftriaxone is the most-common empirical antibiotic used for the community-acquired infections for hospitalized patients in Thailand [12]. Hence, there has been growing concerns about the global emergence of antimicrobial-resistance gonorrhea, which resulted in the establishment of the global Enhanced Gonococcal Antimicrobial Surveillance Program (EGASP) in Thailand [13], [14], [15]. A recent study from the Thai-EGASP demonstrated no isolates of cephalosporin-resistant *N. gonorrhoeae* from a total of 591 isolates during 2015–2016, in contrast to the data from the other surveillance programs in the United States and Europe [15].

Due to the lack of clinical and microbiological data for DGI in Thailand, this study aims to determine the clinical characteristics and the outcomes of the patients with DGI and to compare the gonococcal antimicrobial susceptibility data between 2 decades.

## Methods

### Patients

The study was conducted at KCMH, a tertiary referral hospital and medical school in Bangkok, Thailand, from January 2002 through September 2019. The medical records of all hospitalized DGI patients classified by the International Classification of Diseases, Tenth Revision (ICD-10) codes or the rheumatology consultation registries were reviewed. The patients were excluded if they had insufficient data or alternative diagnoses including crystal-induced arthritis, non-gonococcal septic arthritis, or reactive arthritis. The demographic data, clinical features, laboratory data, treatments, and outcomes were collected. Concurrent other sexually transmitted diseases were defined as hepatitis B infection (positive test of HBsAg), hepatitis C infection (positive test of anti-HCV antibody), Chlamydial infection (positive test of nucleic acid amplification test or culture for *Chlamydia trachomatis*), HIV infection (positive test of anti-HIV antibody), and syphilis (positive test of the serologic test for syphilis). Time to clinical response was defined as the duration from the date of starting of the antibiotic use to the date of the clinical improvement. Complete and partial responses were defined as the complete and partial resolution of fever, joint symptom, and skin lesions prior to discharge, respectively.

The DGI patients were defined as definite and probable DGI accordingly. Definite DGI had at least 1 of 4 clinical characteristic features of DGI (arthralgia, arthritis, tenosynovitis, or dermatitis, which consisted of macule, hemorrhagic papule, pustule, vesicle, bullae, erythema nodosum, or erythema multiforme) with the evidence of gonococcal infection by Gram stain, culture, or nucleic acid amplification tests from blood, synovial fluid, or primary sites of infections (cervix, urethra, rectum, or pharynx). Probable DGI had at least 2 of the 4 clinical characteristic features of DGI with response to treatment to third-generation cephalosporins within 24–48 h, without the evidence of gonococcal infections by the aforementioned methods.

This study was approved by the Chulalongkorn University Institutional Review Board (certificate of approval no. 596/62).

### Statistical analysis

Continuous variables were reported as median, interquartile range (IQR), and range. Categorical variables were reported as numbers and percentages. The association between each dichotomized group with continuous and categorical variables were assessed using the Mann–Whitney *U* test and the Fisher's exact test, respectively. All tests were two-tailed, and the *P*-value of <0.05 was considered as a statistical significance. All statistical analyses were performed using SPSS for Windows, version 22.0 (SPSS Inc., Chicago, IL, USA).

## Results

**Figure 1.**
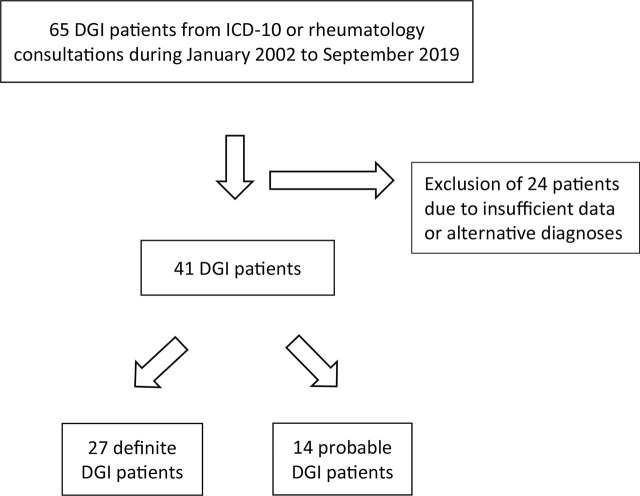
Flow chart of the recruitment of DGI patients. DGI, disseminated gonococcal infection.

All the medical records of 65 hospitalized DGI patients classified by either the ICD-10 codes or the rheumatology consultation registries were reviewed. Of these, 24 patients were excluded from this study due to insufficient data or alternative diagnoses. A total of 41 patients were reviewed and classified into 2 groups. The definite and probable DGI groups consisted of 27 and 14 patients, respectively (**Figure 1**).

The demographic data of the patients with DGI are summarized in **Table 1**. The age of the patients ranged from 16 years to 81 years with the median age of 30 years. Half of the patients (53.7%) were between 15 years old and 30 years old, while about 10% of the patients were >50 years of age, including 3 elderly patients (2 males of the ages 63 years and 66 years and 1 female of the age 81 years). There were slightly more common occurrences among females (58.5%) than among males (41.5%). Comorbidities in patients with DGI were presented in 14 of the 41 patients (34.1%), including systemic lupus erythematosus (14.6%), diabetes mellitus (12.2%), HIV infection (4.9%), and pregnancy (2.4%). No patient with splenectomy was reported. There were no data available for menstruation or terminal complement deficiency. Concurrent sexually transmitted diseases were 5% of hepatitis B infection (1/20 patients), no hepatitis C infection (0/15 patients), 15.4% of Chlamydial infection (2/13 patients), and 14.3% of syphilis (3/21 patients). Patients having a history of antibiotics and immunosuppressive drug use prior to admission were 36.6% and 14.6%, respectively. In addition, 29.3% and 12.2% of the patients had multiple partners and homosexual behavior, respectively.

**Table 1.** Demographic data of patients with DGI

	**Total patients (*n* = 41)**	**Definite DGI (*n* = 27)**	**Probable DGI (*n* = 14)**
Age at diagnosis	30, 20–37, 16–81	33, 22–41, 16–81	22, 19–32, 16–42
Median, IQR, range (years)			
15–30	22 (53.7%)	12 (44.4%)	10 (71.4%)
31–40	10 (24.4%)	8 (29.6%)	2 (14.3%)
41–50	5 (12.2%)	3 (11.1%)	2 (14.3%)
51–60	1 (2.4%)	1 (3.7%)	0
>60	3 (7.3%)	3 (11.1%)	0
Sex			
Male	17 (41.5%)	10 (37%)	7 (50%)
Female	24 (58.5%)	17 (63%)	7 (50%)

DGI, disseminated gonococcal infection.

### Clinical data

The clinical data of the patients with DGI according to the group are summarized in **Table 2**. Of these 41 patients, the median duration of fever, joint pain, and time from the last sexual intercourse prior to presentation were 4 d (IQR 1–6.5, range 1–16), 4 d (IQR 2.5–7.5, range 1–21), and 14 d (IQR 7–24.8, range 1–1,825), respectively. For the presenting symptoms, subjective fever, chills, joint pain, rash, and genitourinary symptoms (dysuria or discharge) were reported in 90.2%, 34.1%, 100%, 26.8%, and 29.3% of the patients, respectively. For the presenting signs, fever (defined by body temperature ≥37.8°C), arthritis, tenosynovitis, skin lesion, and urethral discharge were observed in 43.2%, 92.7%, 63.4%, 39%, and 17.1% of the patients, respectively. Tenosynovitis was the only clinical feature that was significantly different between the definite and probable DGI groups, (51.9% vs. 85.7%, *P* = 0.033).

**Table 2.** Clinical data of the patients with DGI

	**Total patients (*n* = 41)**	**Definite DGI (*n* = 27)**	**Probable DGI (*n* = 14)**	*P* ^*^
Presenting symptoms				
Fever	37 (90.2%)	26 (96.3%)	11 (78.6%)	0.107
Chills	14 (34.1%)	8 (29.6%)	6 (42.9%)	0.494
Joint pain	41 (100%)	27 (100%)	14 (100%)	–
Skin lesions	11 (26.8%)	7 (25.9%)	4 (28.6%)	1.000
Genitourinary symptoms (dysuria or discharge)	12 (29.3%)	9 (33.3%)	3 (21.4%)	0.494
Presenting signs				
Arthritis	38 (92.7%)	25 (92.6%)	13 (92.9%)	1.000
Number				
Median, IQR, range	2, 1–3, 1–14	2, 1–3, 1–14	2, 1–3, 1–7	1.000
Distribution of joint involvement				0.885
Monoarthritis	14 (36.8%)	10 (40%)	4 (30.8%)	
Oligoarthritis	20 (52.6%)	12 (48%)	8 (61.5%)	
Polyarthritis	4 (10.5%)	3 (12%)	1 (7.7%)	
Tenosynovitis	26 (63.4%)	14 (51.9%)	12 (85.7%)	0.033
Skin lesion	16 (39%)	9 (33.3%)	7 (50%)	0.300
Urethral discharge				
Yes	7 (17.1%)	5 (20.8%)	2 (14.3%)	
No	31 (75.6%)	19 (79.2%)	12 (85.7%)	1.000
Missing	3 cases	3 cases	-	

DGI, disseminated gonococcal infection.

* *P* by Fisher's exact test and Mann–Whitney *U* test.

The patterns of joint involvements are summarized in **Table 3**. The median number of joint involvements was 2 joints (IQR 1–3, range 1–14). Monoarthritis, oligoarthritis (2–4 joints), and polyarthritis (5 joints or more) were observed in 36.8%, 52.6%, and 10.5% of the patients, respectively. The commonly involved joints were the wrists (47.4%), the ankles (44.7%), and the knees (36.8%). There was no significant difference of the joint involvement between the definite and probable DGI groups.

**Table 3.** The pattern of joint involvements

	**Number (%)**
Number of joint involvements	
Monoarthritis	14 (36.8)
Oligoarthritis	20 (52.6)
Polyarthritis	4 (10.5)
Site of joint involvements	
Wrist joint	18 (47.4)
Ankle joint	17 (44.7)
Knee joint	14 (36.8)
Shoulder joint	6 (15.8)
Metacarpophalangeal (MCP) joint	6 (15.8)
Elbow joint	5 (13.2)
Proximal interphalangeal (PIP) joint	3 (7.9)
Hip joint	2 (5.3)
Tarsometatarsal joint	2 (5.3)
Sternoclavicular joint	1 (2.6)
Axial and sacroiliac joint	0 (0)
Temporomandibular joint	0 (0)

### Laboratory and radiographic data

Leukocytosis (White blood cells (WBC) ≥10,000 cells/mm^3^) and pyuria (WBC ≥10 cells/high power field on urinalysis) were found in 89.5% (17/19) and 36.8% (7/19) of the patients, respectively. There was no significant difference of the laboratory data between the definite and probable DGI groups.

Of the 23 available synovial fluid analyses, the median WBC and percentage of neutrophils were 25,000 cells/mm^3^ (IQR 8,500–80,000, range 30–123,840) and 91.0% (IQR 80.0–94.6, range 63.0–97.0), respectively. The synovial fluid analyses were classified as noninflammatory (WBC <2,000 cells/mm^3^), inflammatory (WBC 2,000–50,000 cells/mm^3^), septic (WBC 50,000–100,000 cells/mm^3^), and purulent (WBC >100,000 cells/mm^3^) in 8.7%, 47.8%, 30.5%, and 13.0%, respectively. There was no significant difference of synovial fluid analyses between the definite and probable DGI groups.

Of the 11 available plain radiographs at presentation, there was no report of joint destruction. Two patients underwent magnetic resonance imaging (MRI) of the infected joints due to no response to the treatment. The first MRI showed evidences of synovitis, cartilage destruction, and osteomyelitis of the wrist; and the other showed synovitis, bone erosion, osteomyelitis of the hip, and pyomyositis of the adjacent muscle, 5 d and 12 d after starting ceftriaxone, respectively.

### Microbiological data

Synovial fluids were tested for aerobic culture, Gram stain, and polymerase chain reaction (PCR) or 16s-rRNA amplification for *N. gonorrhoeae* in 33, 29, and 3 patients, respectively. Of these, tests yielded positive culture of *N. gonorrhoeae*, positive for Gram-negative diplococci, and detection of *N. gonorrhoeae* nucleic acid in 10 of the 33 (30.3%), 11 of the 29 (37.9%), and 3 of the 3 tested specimens, respectively. There were discrepancies between the methods of bacterial detections. Gram stain was positive in 6 of the 23 (26.1%) culture-negative specimens and PCR or 16s-rRNA amplifications for *N. gonorrhoeae* were positive in 2 of the 3 culture-negative specimens.

Hemocultures were tested in 32 patients and were positive for *N. gonorrhoeae* in 4 of the 32 (12.5%) tested specimens. The other specimens from primary mucosal sites (i.e., cervix, urethra, rectum, or pharynx) were also tested. The number of tested patients and positive specimens according to the site of the specimens are summarized in **Table 4**. Positive cultures of *N. gonorrhoeae* were found in 33.3% (5/15 tests), 18.8% (3/16 tests), 13.3% (2/15 tests), and 9.1% (1/11 tests) from the cervices, urethrae, pharynges, and rectums, respectively. One patient showed positive culture from a pustule. There were no data available on the use of Thayer–Martin medium and the carbon dioxide-generating transport system.

**Table 4.** Microbiological data of the patients with DGI

	**Number of tested patients (%)**	**Number of positive specimens (%)**
Hemoculture	32 (78.0)	4 (12.5)
Synovial fluid for aerobic culture	33 (80.5)	10 (30.3)
Synovial fluid for Gram stain	29 (70.7)	11 (37.9)
Synovial fluid for PCR or 16s-rRNA amplification for *N. gonorrhoeae*	3 (7.3)	3 (100)
Specimen from mucosae for aerobic culture		
Cervical swab	15 (36.5)	5 (33.3)
Urethral swab	16 (39.0)	3 (18.8)
Pharyngeal swab	15 (36.5)	2 (13.3)
Rectal swab	11 (26.8)	1 (9.1)
Pustule for aerobic culture	1 (2.4)	1 (100)

DGI, disseminated gonococcal infection.

Antimicrobial susceptibility tests (ASTs) were performed from the isolates of *N. gonorrhoeae.* All of the 19 isolates were susceptible to ceftriaxone; only 2 of the isolates were tested for the minimal inhibitory concentration (MIC) due to the suspicion of the ceftriaxone resistance, which had the MIC values of 0.004 and 0.016. All of the 7 isolates were susceptible to cefoxitin. However, 17 of the 19 (89.5%) isolates had resistance to ciprofloxacin, tetracycline, and penicillin at the same rate, and 2 of the 19 (10.5%) isolates had intermediate sensitivities to the drugs. No change in the trend of antimicrobial-resistance patterns between 2002–2009 and 2010–2019 were observed.

### Treatments

Of the 39 patients with the available treatment data, all patients were treated with intravenous ceftriaxone followed by outpatient parenteral antibiotic therapies (OPATs) or oral antibiotic therapies according to physicians' decisions. The median duration of total antibiotic therapies among the patients was 14.0 d (IQR 7.0–14, range 7–42). Furthermore, 31 of the 39 (79.5%) and 8 of the 39 (20.5%) patients received a total duration of antibiotic therapy for 7–14 d and >14 d, respectively. In all, 4 of the 39 (10.3%) patients underwent surgeries including the arthrotomy or the arthroscopic debridement of the infected joints because of no response to antibiotic treatments or due to inaccessible arthrocentesis. All patients who underwent surgeries had a median duration of total antibiotic therapies of 35 days (IQR 28–42, range 28–42). The microbial susceptibility tests of those 4 patients with surgical joint drainages were ceftriaxone sensitive in 1 patient and not available in 3 patients. The patients who underwent surgeries had a statistically significant higher median duration of total antibiotic therapies than those who received antibiotics alone (35 d; IQR 28–42 vs 10 d; IQR 7–14; *P* = 0.002). However, no clinical features and laboratory data were significantly different between these 2 groups.

Screenings of *C. trachomatis* by urethral swab cultures or PCR were positive in 2 of the 13 patients (15.4%), but concurrent treatments of chlamydial infection were given in 28 of the 39 patients (71.8%) with doxycycline (71.4%) or azithromycin (28.6%). Serologic tests for syphilis were positive in 3 of the 21 patients (14.3%), and 2 patients were treated with the benzathine penicillin G for late latent syphilis.

### Outcomes

The majority of the patients had good clinical outcomes; 35 of the 41 (85.4%) patients had complete responses prior to discharge. Of those 6 patients with partial responses prior to discharge, 3 of the 6 patients still had the partial responses during the follow-up visits of 2 weeks, while the others missed their follow-ups. No clinical features were significantly different between patients in the complete-response and partial-response groups. The patients with 7–14 d of antibiotic therapies had more statistically significant complete responses than those with >14 d of antibiotic therapies (96.8% vs. 50%; *P* = 0.004). Also, the patients treated by the antibiotics alone had more statistically significant clinical responses within 2 days than those treated by antibiotics and surgeries (66.7% vs. 0%; *P* = 0.024).

Complications occurred in 2 of the 41 (4.8%) patients; 1 patient had osteomyelitis of the wrist, and the other had osteomyelitis of the hip and pyomyositis. Endocarditis, meningitis, perihepatitis, and death were not found in this study.

## Discussion

DGI caused by *N. gonorrhoeae* commonly presents with the rheumatic manifestations of the classic triad of arthralgia or arthritis, tenosynovitis, and dermatitis [1]. DGI occurs in between 0.05% and 1.9% of the gonococcal infections [4, 8, 16]. Due to the paucity of clinical and microbiological data for DGI in Thailand, we conducted a retrospective study of the patients with DGI in KCMH during 2002–2019. As there was no significant difference in the clinical features between the definite and probable DGI groups, except for more patients with tenosynovitis in the probable DGI group, we combined these 2 groups for further analysis and discussion. The age group of DGI in this study tended to be older than in the historical studies [6, 17, 18]. The young adult group (15–30 years of age) accounted for only half of the DGI patients (53.7%), while about 20% of the DGI patients in our study were diagnosed in the middle-aged (40–60 years of age) and elderly patients (>60 years of age). In recent years, there was a report from Australia that the young adult group (15–30 years of age) accounted for 35% of the adult DGI patients, while the middle-aged and elderly patients accounted for 38% of the adult DGI patients [8]. This might reflect the fact that the Thai population is going to be an aging society, and those elderly still have active sexual lives. So, DGI should be included in the differential diagnosis of the septic arthritis in the elderly patients. DGI is 3–4 times more common in female patients [6, 7, 18]. There were slightly more common occurrences among females than among males with a ratio of 1.4:1 in this study. There was a recent study found that there is no gender difference in the DGI patients [8]. Our study showed that common manifestations of DGI were also fever, arthritis, and tenosynovitis which were observed in 90.2%, 92.7%, and 63.4% of the patients, respectively. Oligoarthritis was the most common joint pattern involvement (52.6%), followed by monoarthritis in 36.8% and polyarthritis in 10.5% of the patients. The wrists, ankles, and knees were commonly involved in 47.4%, 44.7%, and 36.8% of the patients, respectively. These findings were comparable with the reports in previous studies [2, 3, 7, 19]. However, the clinical manifestations may be variable among the studies due to the differences in the patients' stages of the diseases and the physicians' examinations [7, 20]. Tenosynovitis was the only clinical feature that was significantly different between the definite and probable DGI groups, as the definition of DGI included tenosynovitis. However, this finding might be altered if different definitions for these 2 groups is used.

Treatments with first-line ceftriaxone were given to all patients. Nonetheless, in this study, 4 of the 41 (9.8%) patients underwent surgeries including open and arthroscopic debridement of the infected joint because of no response to antibiotic treatments or due to inaccessible joint; 2 of these had concurrent acute osteomyelitis, which was diagnosed early by MRI. Although only limited data on the surgical management of the patients with DGI existed, a recent study reported that 41 of the 106 (38.7%) patients underwent surgery. However, the detailed indications are not described [8]. These data were conflicting with the infrequent surgeries performed in the previous studies despite the patients with complications [21], [22], [23]. Whether these changes in the trends of the treatments affect the long-term outcomes remain to be elucidated. Moreover, there were no data available on the testing of complement deficiency in the patients who did not respond to antibiotics. This should be performed especially in those who did not have any comorbid conditions. For the outcomes of patients, the results were comparable to the previous study. The majority of the patients had good clinical outcomes [23], while complications occurred in 4.8% of patients including osteomyelitis and pyomyositis. Endocarditis, meningitis, perihepatitis, and other non-musculoskeletal complications were not observed in this study. The patients who underwent surgeries had longer time to clinical response and longer duration of total antibiotic therapy than those treated by antibiotics alone. These findings were explained by the fact that the surgical group included the patients who did not respond to antibiotics.

In this study, positive cultures from blood, synovial fluid, and other clinical specimens collected from primary mucosal sites in patients with DGI were lower than those reported in previous studies and literature review [3], [5], [6], [7]. This can be explained by the difference in laboratory techniques and the rate of antibiotic use prior to the cultures in the studies. Due to the lack of data on the use of the Thayer–Martin medium and the carbon dioxide-generating transport system, this might explain the lower rate of positive cultures in this study. Despite the usefulness of the nucleic acid amplification tests by PCR or the 16s-rRNA amplification test for the detection of *N. gonorrhoeae* in the selected patients [24], their diagnostic value could not be determined in this study owing to the limited data on the number of tests and the discrepancy in the results compared to the standard culture.

All gonococcal antimicrobial susceptibility results from 19 isolates were susceptible to ceftriaxone, and the majority were resistant to ciprofloxacin and penicillin consistent with the recent report from the Thai-EGASP [15]. These findings confirm that ceftriaxone should remain the first-line treatment of Thai patients with DGI. In the patients with beta-lactam allergy, gentamicin should be the alternative treatment due to the high rate of ciprofloxacin-resistant strains [25]. Spectinomycin is also the alternative option. However, it is currently not available in Thailand [13, 25].

Our study had several limitations. Firstly, the study had a relatively small sample size because of the exclusion of reports with missing data even though the reports were reviewed from 2002 to 2019. This might affect clinical features and outcomes. Secondly, azithromycin susceptibility and ceftriaxone MIC values of *N. gonorrhoeae* isolates were not routinely performed, so we were unable to observe the trend of the antimicrobial-resistance pattern.

## Conclusion

During the past 2 decades, Thai patients with DGI in KCMH still had clinical manifestations similar to the previous studies. However, the ages tend to be older, and there is no gender difference as in the historical studies. Ceftriaxone is still an effective first-line treatment despite the widespread uses of ceftriaxone; the majority of the patients had good clinical outcomes. Despite ceftriaxone being the most common empirical antibiotic for community-acquired infections in Thailand, there is no report of a case with ceftriaxone-resistant *N. gonorrhoeae* in our study. However, due to the high rate of ciprofloxacin- and penicillin-resistant strains, quinolone should not be used as an alternative treatment.
